# When falls become fatal—Clinical care sequence

**DOI:** 10.1371/journal.pone.0244862

**Published:** 2021-01-06

**Authors:** Stacy A. Drake, Sadie H. Conway, Yijiong Yang, Latarsha S. Cheatham, Dwayne A. Wolf, Sasha D. Adams, Charles E. Wade, John B. Holcomb

**Affiliations:** 1 Texas A&M University, College of Nursing, College Station, Texas, United States of America; 2 The University of Texas Health Science Center at Houston School of Public Health, Houston, Texas, United States of America; 3 The University of Texas Health Science Center at Houston, Cizik School of Nursing, Houston, Texas, United States of America; 4 Harris County Institute of Forensic Sciences, Houston, Texas, United States of America; 5 Center for Translational Injury Research, Houston, Texas, United States of America; 6 McGovern Medical School at The University of Texas Health Science Center at Houston, Houston, Texas, United States of America; 7 Department of Surgery, University of Alabama, Birmingham, Alabama, United States of America; John Hunter Hospital and University of Newcastle, AUSTRALIA

## Abstract

**Objectives:**

This study encompassed fall-related deaths, including those who died prior to medical care, that were admitted to multiple healthcare institutions, regardless of whether they died at home, in long-term care, or in hospice. The common element was that all deaths resulted directly or indirectly from injuries sustained during a fall, regardless of the temporal relationship. This comprehensive approach provides an unusual illustration of the clinical sequence of fall–related deaths. Understanding this pathway lays the groundwork for identification of gaps in healthcare needs.

**Design:**

This is a retrospective study of 2014 fall-related deaths recorded by one medical examiner’s office (n = 511) within a larger dataset of all trauma related deaths (n = 1848). Decedent demographic characteristics and fall-related variables associated with the deaths were coded and described.

**Results:**

Of those falling, 483 (94.5%) were from heights less than 10 feet and 394 (77.1%) were aged 65+. The largest proportion of deaths (n = 267, 52.3%) occurred post-discharge from an acute care setting. Of those who had a documented prior fall, 216 (42.3%) had a history of one fall while 31 (6.1%) had ≥2 falls prior to their fatal incident. For the 267 post-acute care deaths, 440 healthcare admissions were involved in their care. Of 267 deaths occurring post-acute care, 129 (48.3%) were readmitted within 30 days. Preventability, defined as opportunities for improvement in care that may have influenced the outcome, was assessed. Of the 1848 trauma deaths, 511 (27.7%) were due to falls of which 361 (70.6%) were determined to be preventable or potentially preventable.

**Conclusion:**

Our data show that readmissions and repeated falls are frequent events in the clinical sequence of fall fatalities. Efforts to prevent fall-related readmissions should be a top priority for improving fall outcomes and increasing the quality of life among those at risk of falling.

## Introduction

Falls from any height are the second leading contributor to unintentional deaths, and The World Health Organization (WHO) rates falls as a global public health priority [1]. In the United States (U.S), one in four persons over the age of 65 years reports falling each year, and, for the past 10 years, fall rates among the elderly have increased by approximately 3% each year [2]. A 2015 U.S. estimate of the economic cost of falls, ranging from minor to serious or fatal, exceeded $50 billion annually [3]. Further illustrating the healthcare economic burden, several studies have identified high readmission rates among those falling [4–7]. From 2007 to 2016, the fall-related mortality of people aged 65 and over has increased by 31% [8]. Despite medical advancements, fall-related mortality has not improved [9]. However, what remains unknown are the root causes of the increasing fall- related mortality.

To address the gap, this study aimed to examine fall related deaths to illustrate the sequence of care in their clinical pathway. This is the first analysis of fall-related mortality to include the full spectrum of fall-related deaths–deaths at the scene (prehospital), deaths during the initial admission (initial acute care setting), and deaths after or during readmissions (post acute care). These pathways demonstrate the complexity in the current healthcare system and provide target points for optimizing care for persons suffering falls.

## Methods

### Data source

Retrospective data included trauma-related deaths occurring in the year 2014 that were within the jurisdiction of the Harris Institute of Forensic Sciences, which includes the Medical Examiner Office, regardless of where the initial injury occurred. All non-natural deaths in Harris County are reported to the medical examiner, including deaths where the initial injury occurred outside Harris County. The initial database (including all mechanisms of injury) contained 1,848 records with information on demographics, mechanism of injury, cause of death, and detailed circumstances surrounding the deaths obtained from medicolegal death investigation, emergency medical service, and hospital records [10]. These data were previously collected to determine a regional trauma preventable/potentially preventable death rate [10,11].

### Study design and sample

**Fig 1:**
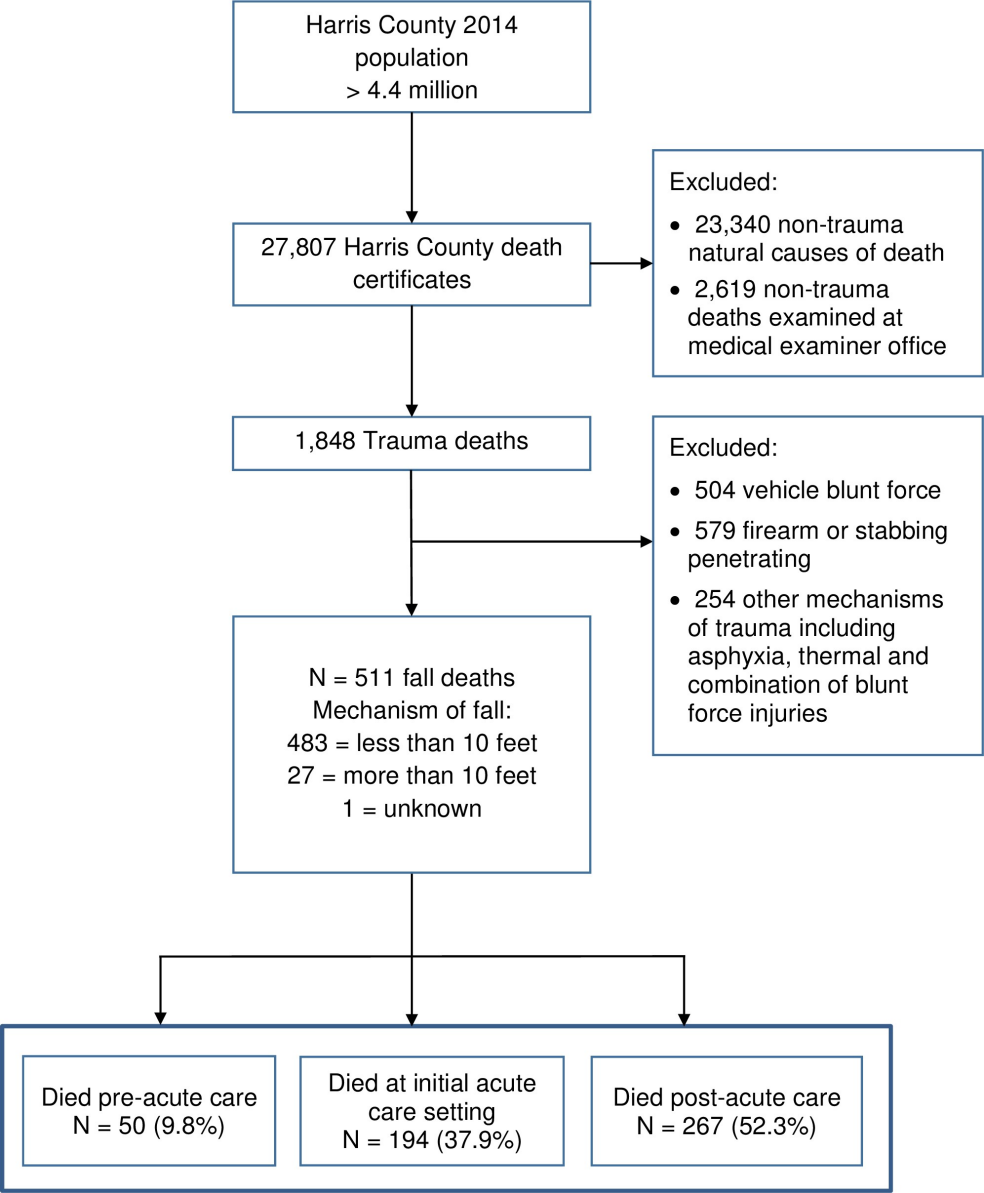
Flow diagram of fall deaths.

The study was a 1-year retrospective analysis of deaths occurring as a result of falls that excluded other causes of death (e.g. other blunt force, firearms, and stabbings); the analytical sample contained 511 fall-related deaths (Fig 1). Because the clinical pathway of falls was a variable of interest, data were examined in relation to where the death occurred, where patients were admitted, and where they were discharged following initial acute care (e.g. nursing home, rehabilitation, home, hospice), the number of documented falls and readmissions, and survival after initial inpatient acute care treatment.

### Measures

Measures included fall-related circumstances, patient demographic characteristics and cohabitation status, and specific confounding variables, including the number of previously reported falls, anticoagulant use, operative procedure, delay in seeking care, prescription or illicit drug use, alcohol use, comorbidities (e.g., hypertension, obesity, diabetes) and preventability determination [11]. Falls were divided into those that occurred from greater or less than 10 feet; the shorter distance falls were largely at ground level.

For this study, clinical pathways are defined as the location of admission and discharge, followed by any subsequent location of readmission, discharge, and place of death. The acute care facilities providing treatment were level I, II, III and IV trauma centers, non-trauma centers, and free-standing emergency departments (ED). This study also included patients who fell and were treated only within nursing homes and primary care physician offices or clinics. The location of deaths were designated as those that occurred (1) in a pre-acute care setting (e.g., at the scene, home or pre-hospitalization), (2) at an acute care setting (e.g., hospital, ED, clinic), or (3) in post-acute care (e.g., post-hospitalization or discharge from facility that initially treated the ultimately fatal injury). Variables related to falls included the number of previous falls, readmission within 30 days after initial care, and survival time after the initial fall. Post-fall survival time was calculated in days, starting from the estimated date of injury (as reported by family/friends or found time) to death date and time. Date of death for persons who died in a pre-acute care setting was actual date of death, if known, or the date the body was found. Injury Severity Score (ISS) measures were calculated by certified injury coders. The scores provided an overall score for estimates of anatomical injury severity [12]. Preventability was defined as opportunities for improvement in care that may have influenced the outcome and refers to death incidences where, given optimal care decisions, anatomic injuries were survivable or when divergence from standard of care directly or indirectly caused the patient’s death [10,11].

### Analysis

Analyses were conducted using IBM SPSS Statistics v22 [13]. Percentages were calculated for fall-related variables by each death location. Univariate analyses for discrete variables were performed using Chi-square test, and Kruskal-Wallis test was used for continuous variables. For all tests, statistical significance was set at the level of α ≤ 0.05.

### Approval

The study was approved by the Committee for the Protection of Human Subjects Institutional Review Boards at both The University of Texas Health Science Center at Houston and Baylor College of Medicine at Houston. All data were de-identified prior to receipt by the research team.

## Results

### Sample characteristics by death location

Demographic characteristics and cohabitation status for the total sample and by death location as well as for selected fall-related variables (e.g., fall height, fall location, survival time) are shown in Table 1. Significance was noted across demographic and fall-related variables.

**Table 1:** Demographic characteristics stratified by death location.

	All Groups n (%)	Pre-acute care^a^ n (%)	Initial acute care^b^ n (%)	Post-acute care^c^ (%)	P Value^d^
**Total**^**e**^	511	50	194	267	
**Preventable/Potentially Preventable**	361 (70.6)	19 (38.0)	120 (61.9)	222 (83.1)	< 0.001
**Age Group, Median (IQR)**	78.6 (67.0–87.2)	57.2 (45.8–69.2)	76.7 (67.9–84.7)	82.2 (71.2–89.5)	< 0.001^f^
Below 45	22 (4.3)	12 (24.0)	7 (3.6)	3 (1.1)	< 0.001
45–64	95 (18.6)	21 (42.0)	31 (16.0)	43 (16.1)	
65 or over	394 (77.1)	17 (34.0)	156 (80.4)	221 (82.8)	
**Sex**					
Male	270 (52.8)	36 (72.0)	109 (56.2)	125 (46.8)	0.002
Female	241 (47.2)	14 (28.0)	85 (43.8)	142 (53.2)	
**Race**					
White	338 (66.2)	33 (66.0)	117 (60.3)	188 (70.5)	0.171
Black	64 (12.5)	9 (18.0)	29 (14.9)	26 (9.7)	
Hispanic	83 (16.2)	6 (12.0)	34 (17.6)	43 (16.1)	
Other^g^	26 (5.1)	2 (4.0)	14 (7.2)	10 (3.7)	
**Mechanism of Injury**					
Fall < 10 feet^h^	483 (94.5)	37 (74.0)	182 (93.8)	264 (98.9)	NA
Fall > 10 feet^i^	27 (5.3)	13 (26.0)	12 (6.2)	2 (0.7)	
Fall from unknown height	1 (0.2)	0 (0.0)	0 (0.0)	1 (0.4)	
**Cohabitation Status**^**j**^					
Lived Alone	106 (20.7)	23 (46.0)	39 (20.1)	44 (16.5)	< 0.001
Not Lived Alone	385 (75.4)	22 (44.0)	146 (75.3)	217 (81.3)	
Unknown	20 (3.9)	5 (10.0)	9 (4.6)	6 (2.2)	
**Location of Fall**					
Residential Property	345 (67.5)	34 (68.0)	133 (68.6)	178 (66.7)	NA
Nursing Home/Rehab/Assisted Living	82 (16.0)	1 (2.0)	26 (13.4)	55 (20.6)	
Public/Commercial Area	72 (14.1)	14 (28.0)	30 (15.5)	28 (10.5)	
Hospital Inpatient	9 (1.8)	0 (0.0)	5 (2.5)	4 (1.5)	
Unknown	3 (0.6)	1 (2.0)	0 (0.0)	2 (0.7)	
**Survival after Injury**^**k**^**, Median (IQR)**	7.9 (1.4–23.0)	0 (0.0–0.0)	2.3 (0.9–7.5)	19.6 (9.5–49.2)	< 0.001^f^
0–24 hours	55 (10.8)	0 (0.0)	52 (26.9)	3 (1.1)	
1 day-30 days	301 (58.9)	0 (0.0)	138 (71.1)	163 (61.1)	
1 month-12 months	93 (18.2)	0 (0.0)	4 (2.0)	89 (33.3)	
> 1 year	12 (2.3)	0 (0.0)	0 (0.0)	12 (4.5)	

^a^Pre-acute care includes those individuals who died prior to arrival to acute care setting, *i*.*e*. dead on scene.

^b^Initial acute care includes those individuals who died during initial hospitalization.

^c^Post-acute care includes individuals who died from injuries or complications of injuries after initial discharge.

^d^Chi-squared test was used to test for differences for variables by death location. The significance level is .05. Because there were no deaths in some categories or small sample size, some statistical analyses’ P values were not applicable.

^e^Five hundred and eleven participants were included in analysis. Percentages for death location were calculated by using the total numbers per location.

^f^Kruskal–Wallis test was used to compare the distribution of age at death and survival time after surgery by death location. The significance level is .05.

^g^Other included Asian and unknown cases.

^h^Fall < 10 feet included fall from ground level.

^i^Fall > 10 feet included falls known to be from a height greater than 10 feet.

^j^Co-habitation with others at time of fatal injurious fall.

^k^Survival time after injury (in days) was from estimated time of injury to time of death. Pre-acute care time of death was the found time. Post acute care survival time was from time of injury to discharge plus time after discharge.

The percentage of overall trauma preventable/potentially preventable deaths was high, with 70.6% of deaths deemed preventable. For those who lived with others, the majority of deaths occurred after discharge (n = 217, 56.4%). For those dying in an initial acute care setting, 52 were either dead upon arrival or within 24 hours following admission, and 76 required a transfer to a higher level of care. For those deaths occurring in post-acute care, the majority occurred within 1 to 30 days of discharge (n = 163, 61.3%).

### Clinical pathways

**Fig 2:**
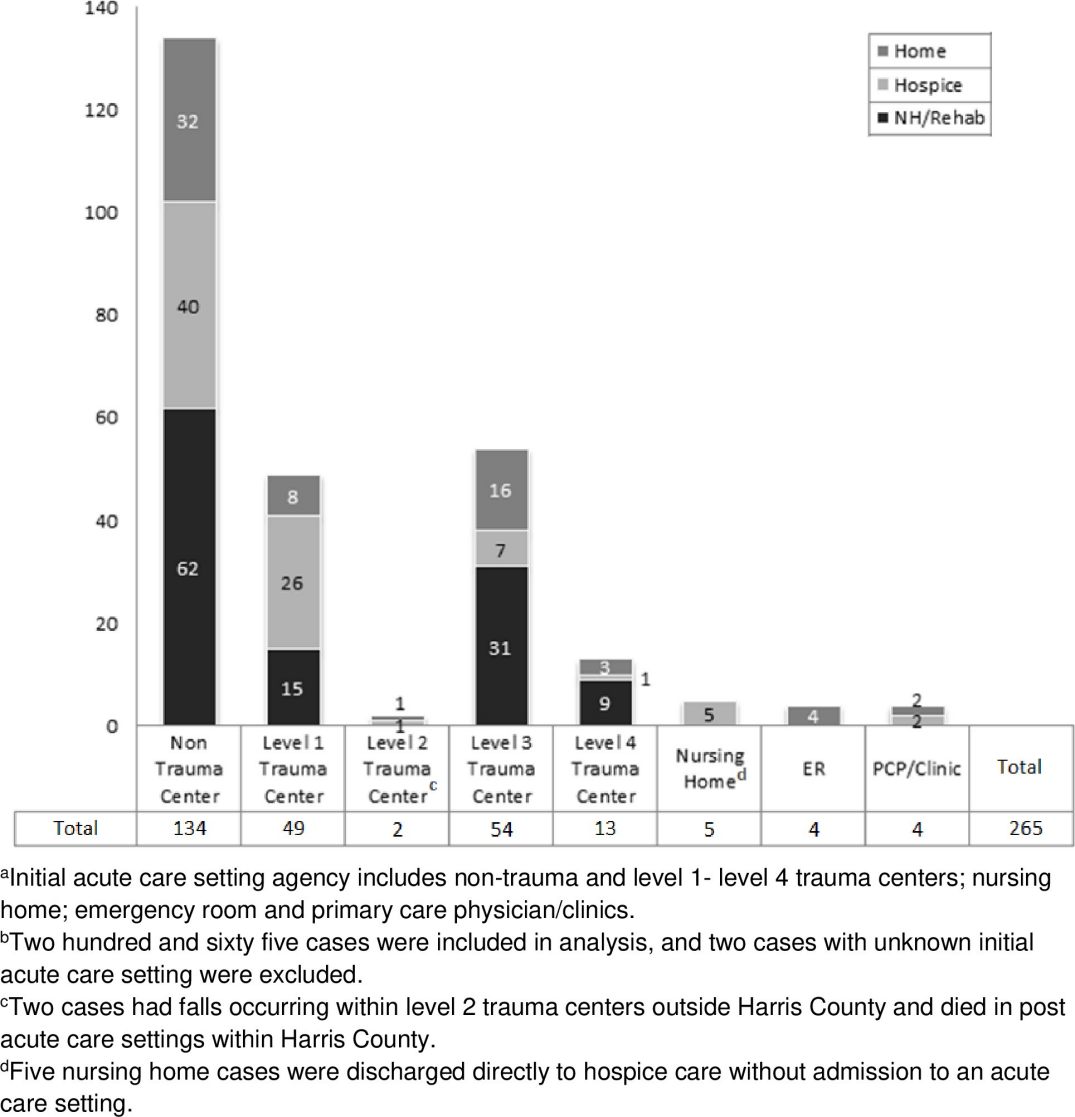
Initial acute care setting and discharge location post-acute care (n = 265).

Fig 2 depicts the location of where 265 post-acute care patients were discharged following their initial care. Of the 265, 117 (44.2%) were transferred for continued care to a nursing home/rehabilitation center, 82 (30.9%) to hospice, and 66 (24.9%) to home. A difference in discharge location occurred between patients treated initially at Level I versus Level III trauma centers and non-trauma acute care facilities: patients treated at Level I facilities were more likely to be discharged to hospice (n = 26, 53.1%) versus patients discharged from level III trauma centers (n = 7, 13.0%). Conversely, patients from level III trauma centers were discharged to nursing home/rehabilitation centers (n = 31, 57.4%) versus patients from level I trauma centers (n = 15, 30.6%).

The data of post-acute care readmissions within 30 days are analyzed from two perspectives: (1) after initial hospitalization (non-trauma and trauma centers), and (2) discharge destination. Of the post-acute care setting patients, 136 (51.3%) had no readmission documented within 30 days, of those 65 (47.8%) were initially treated in non-trauma centers; 33 (24.3%) in Level I trauma centers; and 25 (18.4%) in Level III trauma centers. Overall, 129 (48.7%) of the post-acute care deaths had at least one documented readmission within 30 days, albeit the largest group (n = 99, or 76.7%) had one readmission prior to death, with 57 (57.6%) occurring after initial hospitalization in a non-trauma center. The majority of readmissions occurred within/between different agencies. For example, one patient may have been discharged from a Level I trauma center to a nursing home but readmitted into a non-trauma center within a different system. With regard to the discharge locations, 37 (27.2%) of the 136 patients with no readmission were sent into nursing home/rehabilitation centers, 20 (14.7%) were discharged to residences, and 79 (58.1%) were discharged to hospice. Of the 117 patients discharged to a nursing home/rehabilitation, 80 (68.4%) had at least one readmission within 30 days. Of the 82 discharged to hospice, 79 (96.3%) had no readmission, and 3 (3.7%) had one documented readmission. Of the 66 discharged back to the home residence, 46 (69.7%) had at least one documented readmission.

### Confounding variables

Counts and percentages for potentially confounding variables (number of previous falls, anticoagulant use, presence of alcohol, prescription or illicit drug uses, injury severity scores, anatomic location of injury, and delay in seeking care), are shown in Table 2. Significant differences were found across death locations for number of previous falls. Among all fatal falls, 249 (48.7%) had no documented history of previous falls; however, 216 (42.3%) had a history of one documented fall prior to the final fatal fall, and 136 (63.0%) of those died in the post-acute care setting. Of those falling multiple times (n = 31 or 6.1%), 25 (80.6%) died in the post-acute care setting.

**Table 2:** Confounding variables stratified by death location.

	All Groups n (%)	Pre-acute care^a^ n (%)	Initial acute care^b^ n (%)	Post-acute care^c^ n (%)	P Value^d^
**Total**^**e**^	511	50	194	267	
**Number of Previous Falls**^**f**^					
1	216 (42.3)	10 (20.0)	70 (36.1)	136 (50.9)	
2	21 (4.1)	2 (4.0)	4 (2.1)	15 (5.6)	
3	8 (1.6)	0 (0.0)	0 (0.0)	8 (3.0)	< 0.001
4	1 (0.2)	0 (0.0)	0 (0.0)	1 (0.4)	
10	1 (0.2)	0 (0.0)	0 (0.0)	1 (0.4)	
None	15 (2.9)	6 (12.0)	4 (2.0)	5 (1.9)	
Unknown^g^	249 (48.7)	32 (64.0)	116 (59.8)	101 (37.8)	
**Anticoagulant Use**^h^	197 (38.6)	7 (14.0)	96 (49.5)	94 (35.2)	< 0.001
**Delay in Seeking Care**	76 (14.9)	4 (8.0)	38 (19.6)	34 (12.7)	0.044
**Withdrawal of Care**^**i**^	302 (59.1)	NA	129 (66.5)	173 (64.8)	NA
**Presence of Alcohol**	31 (6.1)	18 (36.0)	13 (6.7)	0 (0.0)	NA
**Presence of Prescription Drug**	14 (2.7)	5 (10.0)	2 (1.0)	7 (2.6)	NA
**Presence of Illicit Drug**	7 (1.4)	2 (4.0)	4 (2.1)	1 (0.4)	NA
**ISS Score, Median (IQR)**	10 (9.0–25.0)	25 (10.0–40.0)	25 (10.0–26.0)	9 (9.0–13.0)	< 0.001^j^
**Anatomical Location**					
Cranial	169 (33.1)	13 (26.0)	95 (49.0)	61 (22.8)	
Vertebral	33 (6.5)	5 (10.0)	8 (4.1)	20 (7.5)	
Thoracic	7 (1.4)	0 (0.0)	2 (1.0)	5 (1.9)	
Abdominal	3 (0.6)	0 (0.0)	2 (1.0)	1 (0.4)	
Pelvic	10 (2.0)	0 (0.0)	1 (0.5)	9 (3.4)	NA
Upper Extremity	23 (4.5)	1 (2.0)	4 (2.1)	18 (6.7)	
Lower Extremity	136 (26.6)	3 (6.0)	24 (12.4)	109 (40.8)	
Multiple	130 (25.4)	28 (56.0)	58 (29.9)	44 (16.5)	

^a^Pre-acute care includes those individuals who died prior to arrival to acute care setting, *i*.*e*. dead on scene.

^b^Initial acute care includes only those individuals who died during initial acute care.

^c^Post-acute care includes individuals who died from injuries or complications of injuries after their initial discharge.

^d^Chi-squared test was used to test differences among categories among variables by death location. The significance level is .05. Because there were no deaths in some categories or small sample size, some statistical analyses’ P values were not applicable.

^e^Five hundred and eleven participants were included in analysis. Percentages for death location were calculated by using the total numbers per location.

^f^Cases with previous fall history reported by family or friends members. Chi-squared test was used to compare individuals with history of fall versus no previous fall history or unknown.

^g^Two hundred and forty nine Cases with unreported or unobserved falls.

^h^Included reported or documented anticoagulant use prior to injury.

^i^Included hospice patients (n = 96) and deaths of which the final decision between family and provider was palliative or comfort care measures.

^j^Kruskal–Wallis test was used to compare the distribution of ISS by death location. The significance level is .05.

### Injury severity scores and comorbidities

As shown in Table 2, the median ISS were higher for those whose deaths occurred in pre-acute and initial acute care settings (Median ISS = 25) than in post-acute care settings (p-value <0.001). Pre-existing comorbidities and death location are shown in Table 3. Among the 511 fall-related deaths, significant differences among death location were found for hypertension 366 (71.6%), diabetes 162 (31.7%), dementia 138 (27.0%), and cardiac arrhythmia 133 (26.0%).

**Table 3:** Comorbidities stratified by death location.

	All Groups n (%)	Pre-acute care^a^ n (%)	Initial acute care^b^ n (%)	Post-acute care^c^ n (%)	P Value^d^
**Total**^**e**^	511	50	194	267	
**Hypertension**	366 (71.6)	21 (42.0)	142 (73.2)	203 (76.0)	< 0.001
**Atherosclerotic cardiovascular**	199 (38.9)	23 (46.0)	75 (38.7)	101 (37.8)	0.551
**Diabetes**	162 (31.7)	7 (14.0)	57 (29.4)	98 (36.7)	0.005
**Dementia**	138 (27.0)	3 (6.0)	30 (15.5)	105 (39.3)	< 0.001
**Cardiac Arrhythmia**	133 (26.0)	3 (6.0)	54 (27.8)	76 (28.5)	0.003
**Congestive Heart Failure**	121 (23.7)	6 (12.0)	50 (25.8)	65 (24.3)	0.116
**Chronic Pulmonary Disease**	90 (17.6)	9 (18.0)	32 (16.5)	49 (18.4)	0.872
**Alcohol Abuse**^**f**^	80 (15.7)	20 (40.0)	25 (12.9)	35 (13.1)	< 0.001
**Thyroid disease**	70 (13.7)	2 (4.0)	15 (7.7)	53 (19.9)	< 0.001
**Cerebrovascular**	68 (13.3)	1 (2.0)	20 (10.3)	47 (17.6)	0.003
**Liver Disease**	58 (11.4)	12 (24.0)	17 (8.8)	29 (10.9)	0.010
**Cancer**	55 (10.8)	4 (8.0)	20 (10.3)	31 (11.6)	0.727
**GERD**	55 (10.8)	2 (4.0)	13 (6.7)	40 (15.0)	0.005
**Renal Disease**	53 (10.4)	1 (2.0)	19 (9.8)	33 (12.4)	0.083
**Anemia**	45 (8.8)	0 (0.0)	12 (6.2)	33 (12.4)	NA
**Arthritis**	37 (7.2)	1 (2.0)	16 (8.2)	20 (7.5)	0.307
**Depression**	35 (6.8)	5 (10.0)	8 (4.1)	22 (8.2)	0.146
**Obesity**	33 (6.5)	4 (8.0)	10 (5.2)	19 (7.1)	0.627
**Peripheral Vascular Disease**	23 (4.5)	1 (2.0)	8 (4.1)	14 (5.2)	0.567
**Valvular Disease**	22 (4.3)	2 (4.0)	7 (3.6)	13 (4.9)	0.800
**Other Substance Abuse**^**g**^	11 (2.2)	3 (6.0)	1 (0.5)	7 (2.6)	NA

^a^Pre-acute care includes those individuals who died prior to arrival to acute care setting, *i*.*e*. dead on scene.

^b^Initial acute care includes individuals who died during initial acute care.

^c^Post-acute care includes individuals who died from injuries or complications of injuries after discharge.

^d^Chi-squared test was used to test for differences among comorbidities by death location. The significance level is .05. Because there were no deaths in some categories or small sample size, some statistical analyses’ P values were not applicable.

^e^Five hundred and eleven participants were included in analysis findings are not mutually exclusive.

^f^Determined based upon clinical history, family interviews, or postmortem findings.

^g^Determined based upon clinical history or family interviews.

## Discussion

The data supports a different way of looking at death by falls, that of examining typical patient progression from initial medical care to death in a post-acute care setting (e.g., resident, long term care/assisted living or rehabilitation center, hospice facility or palliative care). Whereas traditionally falls are related to age progression [2,8], our study findings suggest patient survivability of an initial fall is influenced by factors inherent in type and level of acute and post-acute care received. From this viewpoint, the finding that almost three quarters of deaths by injuries sustained in falls (70.6%, 361 of 511) were considered preventable or potentially preventable raises the question as to what could have been changed to effect a better outcome. This high percentage suggests that points for intervention exist along the continuum from fall to death that may have reduced readmissions or extended survivability, had timely interventions been used or available. This shifts the lens in which we address prevention of falls to examining circumstances and conditions that either hasten or hinder recovery as well as possibly preventing the reoccurrence of falls.

In reference to data relating to individual deaths, forensic pathologists in the Harris County Medical Examiner’s Office determined the cause and manner of death, based on accepted practice [14], and certified death by injuries from falls as non-natural (e.g., accident, suicide or homicide), which eliminated deaths where natural disease was the sole cause of death.

In our study, 483 (94.5%) patient falls occurred at ground level, for example, getting out of bed, transitioning from sitting to standing, or walking. Ground level falls are the primary cause of injury sustained by patients admitted into trauma centers, and these falls account for over 40% of admissions in most centers [15]. Hospitals are aware of the high risks of falls for inpatients [15,16]. During an inpatient stay, patients may require stricter monitoring to minimize potentially preventable injuries from reoccurring falls. Discharge planning should include review of a patient’s prescription medications to assure that none contribute to gait unsteadiness. If a patient is discharged home, interventions could also include environmental scans for safety, and if discharged to rehabilitative care, the use of technology to monitor motor balance [15].

For those who death occurred in a post-acute care setting (n = 222 of 267), we traced patient movement through clinical pathways, where the most common pathway was from residence to acute care, discharge to rehabilitative care, and return to residence. An alternate pathway, mainly dependent on severity of injury sustained from the initial fall, was from hospital to hospice or to some other palliative care option. Additionally, our findings highlighted the large number of facilities providing post-acute care: those 267 patients had been involved with 440 admissions before death in a post-acute care setting. Some reasons for this high number of facilities were transfer to a facility providing additional skilled care or transfer from rehabilitative care to hospice or to some other form of palliative care.

A number of deaths occurred within the initial treating facilities, including nursing homes (n = 194). Factors possibly contributing to inpatient deaths were delay in seeking help and higher injury severity. Delay to initial treatment has implications as well, since poor outcomes have been identified for patients presenting for care after more than four hours after injury [17]. Reasons for delay in seeking care may include lack of access to a medical facility, personal dislike for seeing a physician, and perception of no immediate clinical change after fall. Another factor for delay could be due to care-giver abuse or neglect [18]. The extent of injuries, as indicated by injury severity scores, were highest (ISS = 25; a score of 15 is considered major trauma [12]) for those who died before seeking medical care and for those who died in the initial acute care setting. In this study, injury was generally associated with cranial and lower extremity injuries; cranial injuries predominated in the group of patients who died before or during the initial acute care setting.

The individuals with the highest ISS were those that fell from heights, where they suffered injuries occurring over multiple body/cavity regions. These were predominately adult patients who died in the pre-acute care setting (scene deaths) or were dead upon arrival. Mitigating steps for fall intervention of the pre-acute care deaths will be challenging because of the multiplicity of precipitating factors and confounders. In this study, fatal falls at ground level were most likely to be male individuals (n = 36 vs. n = 14 for females) who lived alone. In this group, chronic alcoholism was identified; but this could have increased the risk for falling and contributed to a delay in calling for assistance.

For patients discharged after initial hospitalization, more patients were transferred to a nursing home or rehabilitation center (n = 117) than to hospice (n = 82) or to a residence (n = 66). This progression from acute care to post-acute care was most likely to be from a Level 1 trauma center or non-trauma facility, and elderly patients, especially non-ambulatory patients with comorbidities, were the most likely candidates for hospice. Post-acute care patients discharged to hospice included those with a diagnosis of dementia, which is a known risk factor for poor surgical outcome recovery [19]. Patients in Level III trauma centers (n = 31) with less severe injuries were more likely to be transferred to a facility for rehabilitation and were least likely to have hospital readmissions. However, we found that 39% (n = 76/194) required a transfer for a higher level of care, either to a Level I trauma center or to a facility with advanced surgical services.

In this sample, almost half of those in a post-acute care setting were readmitted within hours to 30 days after initial hospitalization (n = 129, 48.7%), and average survival times from the initial fall were less than a month. Patients were readmitted due to fall-related complications (e.g., pneumonia, sepsis, multi-system organ failure) or from a fall reoccurrence. This high readmission may indicate a failure in provision of healthcare, whether due to clinical decisions or patient/family non-compliance with medical directives or medication issues. Initiatives to decrease readmissions and optimize individualized, multifactorial fall prevention interventions involving patient and family should be a priority [15]. In addition, alternate post-care settings could be made available, as in palliative care and home-based physical therapies. Appropriate level of care may include level I trauma center(s), hospitals with geriatric capabilities, or facilities with advanced neurosurgical and cardiac specialties [15]. Additionally, EMS providers have an opportunity to consider reviewing pre-hospital triage guidelines to ensure that fall patients are initially transported to the appropriate level of care.

Inpatient falls are key indicators of quality of care and can cause severe consequences for patients, families, and facilities [20]. However, inpatient fatal falls were a small percentage within this cohort (2.5%). Measures used to prevent inpatient falls, if inappropriately implemented, may restrict mobility [20,21]. One study found that hospitalized elderly, capable of walking independently, spent an average of 20 of 24 hours in bed [22]. Decreased mobility predisposes elderly patients to loss of muscle mass and functional decline [23,24]. Failure to recognize mobility- restriction morbidity in hospitalized patients may result in patients transitioning back to their residence, where their functional decline predisposed them to subsequent falls. In addition, 16% of the recurring falls occurred within nursing homes, assisted living or rehabilitation centers. This suggests that strategies to prevent fall reoccurrence are needed. A systematic review of barriers and facilitators for fall prevention in residential care facilities suggested that use of a multidisciplinary team to implement individualized interventions could effectively reduce fall reoccurrence [25].

### Fall prevention

Medical records do not provide a clear account of why an individual fell, even when an eye witness to the fall was interviewed by the caregiver. Possibilities for why fatal falls occurred are endless and include accidents and suicides as well as personal, medical, socio-economic, and environmental aspects, the diversity of which complicates preventive measures [26]. However, timely interventions could ameliorate recovery and lessen readmissions and fall reoccurrences.

An example of a fall prevention strategy is that emergency medical service providers could effectively take the lead to prevent the first fall through “lift assist” calls. During these calls, the responding team could quickly conduct an environmental assessment and offer real time referrals for community or home based injury prevention programs. They may also help those who live alone with medication compliance [27], especially in reference to cardiac and anti-cholinergic agents, since compliance or non-compliance with these medications may be associated with syncope or gait instability [28]. Moreover, in this sample, a majority of patients suffered from hypertension and cardiovascular problems and often the fall was a precursor of worsening health.

Public health initiatives can leverage evidence-based community programs for mentoring health and providing therapies for increasing physical activity and improving balance [29]. Personal alert systems are of benefit to those aging at home. After a non-fatal initial fall, discharge planning for those released to home should include patient and family/caregiver understanding of possible fall prevention strategies, including modification of lifestyle behaviors, to prevent reoccurrence of falls [15], and institution of personal alert systems for when falls do occur.

The burden for preventing reoccurring falls is the responsibility of both the individual and their health care-givers in all phases of care (e.g., pre-hospital, acute care, and discharge to locations responsible for continued care). To date, standardized protocols for assessing fall risk, tailoring personalized fall prevention strategies, and evaluating success of strategies are not universally in place [15]. Additionally, no one strategy is known for reducing readmissions or death deaths [30,31].

### Strengths and limitations

Strengths of this study included the availability of high-quality documentation of all fall-related deaths occurring a one-year period, regardless of how many healthcare institutions were involved or whether the patient was even treated for the injuries. Based on medicolegal death investigation data, we traced the fatal fall pathway from injury to death. Because the sample was limited to deaths in one county (albeit the county of over 4 million population) generalizability may be limited. Although some findings were similar to other data analysis and results from other study samples [2,6], our findings produced a different way at looking at preventability of death by injuries, that of a progression from fall to death. Limitations are the lack of information on non-fatal falls for comparative purposes. Further study incorporating analogous data from other counties and in subsequent years would allow for inferences about geographic differences and temporal trends.

## Conclusion

Our data show that readmissions and repeated falls are frequent events in the clinical sequence of fall fatalities. Therefore, efforts directed at readmission and preventing subsequent falls should be a top priority for improving outcomes and increasing the quality of life.
